# Graham Patch Repair of a Prepyloric Ulcer Complicated by Recurrent Abdominal Abscesses and Leukocytosis: A Case Report

**DOI:** 10.7759/cureus.54646

**Published:** 2024-02-21

**Authors:** Ehizele P Itama, Kelly Tran, Pratik Patel, Yash Patel, Anahita Saifollahi, Nicole Dushkin, Frederick Tiesenga

**Affiliations:** 1 Surgery, American University of Barbados, Saint Michael, BRB; 2 Surgery, Washington University of Health & Science, San Pedro, BLZ; 3 Surgery, St George's University School of Medicine, St. George's, GRD; 4 General Surgery, West Suburban Medical Center, Chicago, USA

**Keywords:** omental patch repair, asa score, peptic perforation, graham patch repair, intraabdominal abscess, laparoscopic modified graham patch repair, pyloric ulcer

## Abstract

This case report describes the clinical course of a 51-year-old Caucasian woman with a history of anemia who presented to the emergency department with worsening diffuse abdominal pain and weakness two days after dental surgery. The patient's condition rapidly deteriorated, manifesting as tachycardia, diaphoresis, and a peritonitic abdomen. A CT scan revealed a perforated gastric ulcer, prompting emergent laparoscopy, Graham patch repair, and abdominal washout. Postoperatively, the patient developed leukocytosis, and imaging indicated the formation of an abscess. Despite initial attempts at percutaneous drainage, a subsequent exploratory laparotomy was performed. The patient's leukocytosis eventually resolved, and she was discharged after 21 days with outpatient follow-up.

The discussion delves into the declining incidence of peptic ulcer disease but a constant rate of complications, emphasizing the role of factors such as nonsteroidal anti-inflammatory drug use. The diagnostic approach using CT scans in suspected perforated peptic ulcers is highlighted. The study also explores risk stratification scoring systems, with a preference for operative management. The laparoscopic omental patch repair (Graham patch) is discussed, citing its safety and efficacy. The case presented an uncommon occurrence of failed primary percutaneous abscess drainage, leading to subsequent surgical drainage. The discussion concludes by noting variables that may contribute to drainage failure and emphasizes the need for further research to understand such complications.

## Introduction

A peptic ulcer occurs when there is a disruption in the inner lining of the digestive tract due to inflammation or irritation. Peptic ulcers consist of gastric ulcers that take place in the stomach and duodenal ulcers that take place in the duodenum of the small intestine. The risk factors include Helicobacter pylori, nonsteroidal anti-inflammatory drugs (NSAIDs), steroids, alcohol, caffeine, smoking, stress, Zollinger-Ellison syndrome, Crohn’s disease, and cirrhosis [1]. A patient with a peptic ulcer presents with abdominal pain, burning, vomiting, belching, poor appetite, weight loss, bleeding, and perforation. To confirm the diagnosis of the ulcer, an endoscopy, or barium swallow, is performed. A peptic ulcer that occurs in H. pylori can be diagnosed by a urea breath test, detecting blood antibodies, or a stool antigen test. To manage the peptic ulcer, lifestyle modification is advised by decreasing the use of NSAIDs, steroids, caffeine intake, smoking cessation, and starting proton pump inhibitor (PPI) drugs [1,2]. PPIs decrease the production of stomach acid, thus preventing further damage to the inner lining of the digestive tract mucosa. Peptic ulcers caused by H. pylori can be prevented by using PPIs, amoxicillin, clarithromycin, and metronidazole [2-4]. In the last decade, the rate of peptic ulcer disease (PUD) has decreased due to the effectiveness of medications and rationalizing the use of NSAIDs [4]. Prolongation of the peptic ulcer leads to other complications, including GI bleeding, perforation of the GI tract, peritonitis, gastric outlet obstruction, and possibly gastric cancer.

Graham patch is a surgical technique that is used to close the perforation using a well-vascularized omentum. The modified technique was employed in this case which involves a first layer of knot along the perforation before the second knot which is made over the omentum. After securing the Graham patch over the perforation, the site is inspected using irrigation fluid and injecting air into the patient’s nasogastric tube. The absence of an air bubble indicates an intact repair [5]. There are some complications associated with Graham patch repair including postop infection, abscess, paralytic ileus, gastric outlet obstruction, necrosis, and postoperative leaks. 

We present a case of a 51-year-old woman with a past medical history of anemia who presented with diffuse abdominal pain, weakness, nonbilious vomiting, chills, tachycardia, and clammy skin. A CT scan revealed free air and fluid in the abdomen due to intestinal perforation. Later, exploratory laparotomy was performed, and Graham patch repair surgery was conducted, which led to a complete resolution of the patient’s symptoms.

## Case presentation

**Figure 1:**
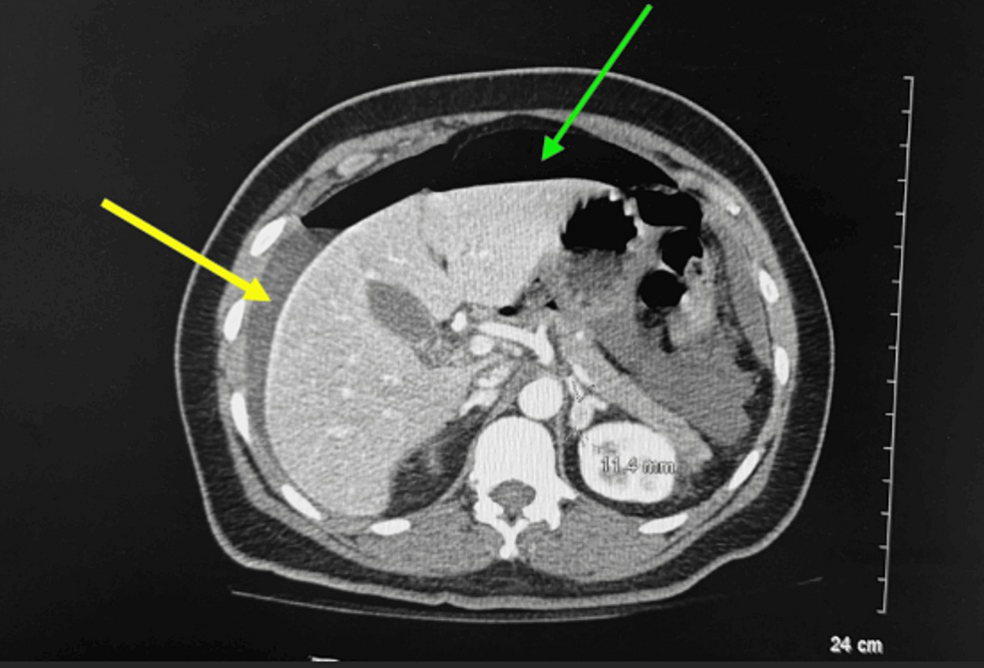
CT scan of the pelvis and abdomen with IV contrast showing free intraperitoneal air (represented by the green arrow) and free fluid (represented by the yellow arrow) suspicious of bowel perforation with focal wall thickening along the gastric pylorus. Findings suspicious for CT hypoperfusion.

A 51-year-old Caucasian female status post dental surgery two days ago with a significant past medical history of anemia presented to the emergency department for diffuse abdominal pain and weakness for two days. She reported associated nausea, nonbilious vomiting, and chills. She was tachycardic with heart rates up to the 130s, diaphoretic, and pale with clammy skin and a peritonitic abdomen. A CT scan with intravenous (IV) contrast of the abdomen and pelvis showed intestinal perforation due to free air and fluid in the abdomen (Figure 1), not thought to be of a hemorrhagic origin. The patient was started on an antibiotic, piperacillin/tazobactam. A diagnostic laparoscopy was performed and revealed a perforated gastric ulcer in the pre-pylorus (distal stomach). A Graham patch repair of the perforated ulcer was performed with abdominal washout and two Jackson-Pratt drains were placed. Large collections of bilious fluid found within the abdomen were cultured. The patient tolerated the procedure well and was put empirically on piperacillin/tazobactam and fluconazole while being admitted to the ICU in stable condition. 

**Figure 2:**
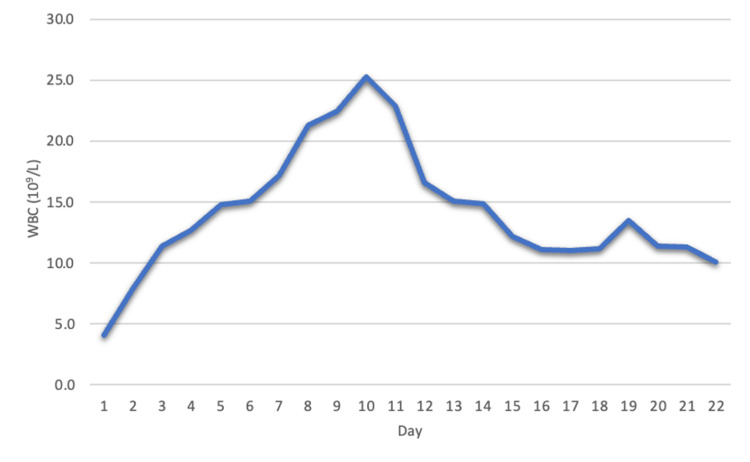
Line graph showing the increasing rate of leukocytosis at the start and then decline toward the end of hospitalization.

**Figure 3:**
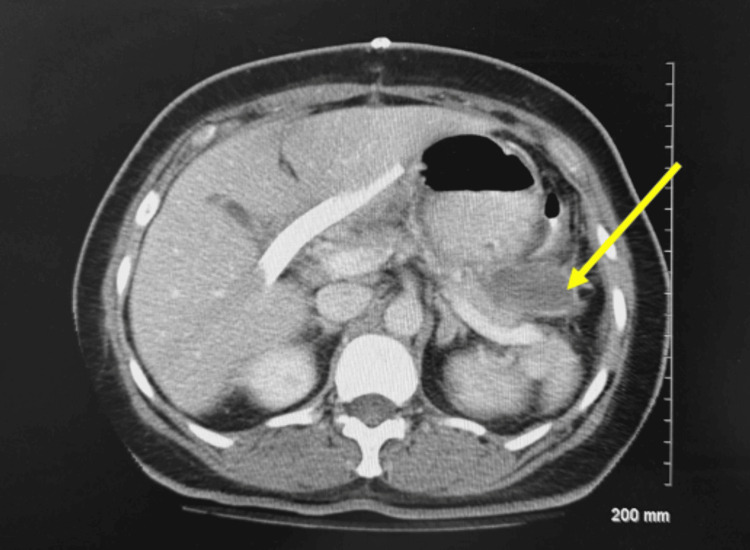
CT Abd/Pelvis with IV contrast on POD 13 showing a fluid collection of 4.7cm in diameter (as represented by the yellow arrow) with an enhancing rim and a small outpouching anteriorly and superiorly that contains a gas bubble. That segment of collection is 14 mm in diameter and consistent with a well-defined abscess.

**Figure 4:**
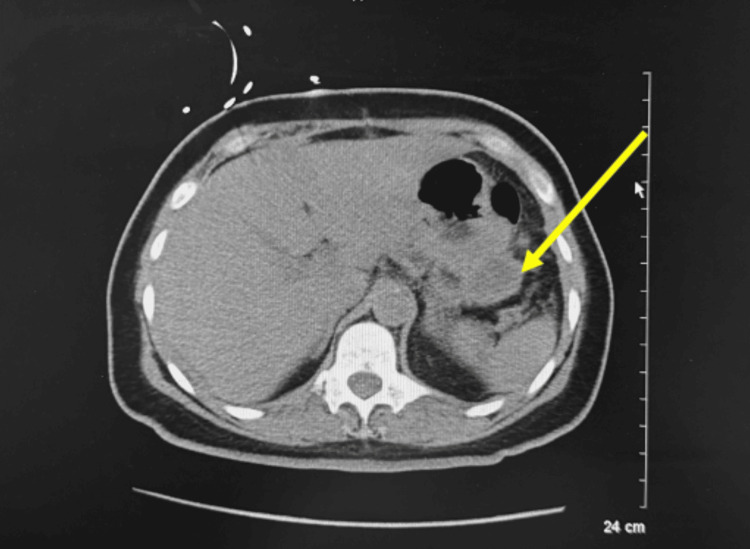
CT Abd/Pelvis with IV contrast on POD 18 showing a significant decrease in size measuring 2.1 x 3.7cm (as represented by the yellow arrow).

The patient then had an uptrend in WBCs (Figure 2) (Table 1) which led to a repeat CT scan that revealed fluid in the left upper quadrant along the greater curvature of the stomach. IR paracentesis was completed and yielded approximately 100mL of serous fluid. The patient was started on IV vancomycin and anidulafungin. Cultures from the graham patch repair showed Citrobacter and the patient underwent exploratory laparotomy with abdominal washout which revealed fibrinous exudate within the lesser sac posterior to the stomach and behind and deep to the uterus in the posterior cul-de-sac. A repeat CT scan showed a 4.7cm fluid collection attached to the gastric fundus with an enhancing rim (Figure 3). Aspiration of the gastric fundus abscess was performed. Culture was unable to yield results due to an insufficient amount of fluid. Repeat aspiration was considered but not completed due to improvements in the leukocytosis and a repeat CT scan that revealed a significant decrease in abscess size (Figure 4). The patient was discharged and to follow up with the surgical team in outpatient for an esophagogastroduodenoscopy. 

**Table 1:** Table showing the increasing rate of leukocytosis post-op s/p Graham patch repair with abdominal washout and decline toward the end of hospitalization.

Day	White Blood Cells (10^9^/L)	Hemoglobin (g/L)	Hematocrit (L/L)
1	4.1	13.6	41.4
7	17.2	8.8	25.9
14	14.9	8.6	26.4
21	11.3	9.6	28.8

## Discussion

The incidence and prevalence of PUD have declined in recent years, but its rates of complications, most commonly hemorrhage or perforation, have remained constant, which does not correlate with the decline of uncomplicated ulcers [6]. This can be attributed to the increase in the use of ASA and NSAIDs [7]. Also, the successful management of Helicobacter pylori, one of the causes of peptic ulcers, plays a role as well. The other main cause is the use of NSAIDs. H. pylori infection is usually present in duodenal and gastric ulcers, but not prepyloric ulcers [8]. This happens to apply to our patient who presented with a prepyloric ulcer perforation but a negative H. pylori test. Although perforation is a complication of PUD, it is less common than bleeding, it is the most common indication for an emergent operation and is responsible for 40% of ulcer-related deaths [9].

Patients with a suspected perforated peptic ulcer used to undergo an upright X-ray of the abdomen and chest to detect the presence of free abdominal air. Now, CT scans are recommended and the most helpful due to their 98% diagnostic accuracy rate and their quick way of excluding acute pancreatitis [10,11]. In cases where there is a suspicion of perforated peptic ulcer, but imaging is negative for free air, imaging with water-soluble contrast is suggested [12]. 

In determining the mortality and morbidity of perforated peptic ulcer postsurgery, there are several risk stratification scoring systems like ASA, PULP, and Boey score. A study performed in the TU teaching hospital in Nepal concluded that the Boey scoring system had better accuracy than the other scoring systems [13]. Boey scoring is made up of three factors: concomitant severe medical illness, preoperative shock, and duration of perforation >24 hrs. If positive, each factor scores a point. Scores of 2 and above have mortality and morbidity rates of 33% and 75% respectively [14].

 With operative management preferred to nonoperative management for perforated peptic ulcers, emergent surgery consultation is required. They are surgical emergencies with a variety of surgical options, but Graham patch repair remains the gold standard of preferred surgical treatment modality. The omental patch, also referred to as the Graham patch, was utilized in our patient’s surgical repair laparoscopically. This involved maneuvering the omentum to the perforation site and securing the site with 3-4 interrupted silk sutures [15]. A 2014 article aimed to study the safety and efficacy of laparoscopic patch repair and found that in 15 patients, 14 were successful, and one had a postoperative complication [16]. Also, it showed a longer operation time and fewer requirements for postoperative analgesics with laparoscopic compared to conventional open patch repair. There was no statistical difference in the hospital duration [16]. 

Postoperative sepsis is a critical illness that requires early detection to prevent death. A 2022 study was done to identify some risk factors in the development of postoperative sepsis which included ages >65 years old, ASA score >2, and associated comorbidities [17]. Postoperative intra-abdominal abscesses can arise and be successfully treated by percutaneous abscess drainage. It uses imaging guidance to place a needle or catheter through the skin into the abscess to drain. An intra-abdominal abscess can be complicated by recurrence. This is uncommon and can predict a poor prognosis. A study conducted by Gervais et. al focused on the incidence and outcomes of secondary percutaneous abscess drainages after successful primary percutaneous abscess drainages. They discovered that abdominal abscesses requiring secondary drainage were postoperative. With regard to location, repeated percutaneous abscess drainages were common in the abdomen or pelvis. In a total of 956 patients, 4.9% of abscesses required secondary percutaneous abscess drainage, and 91% were successful in clearing the abscess contents [18]. In cases where percutaneous drainage is not improving the outcome, sepsis has occurred or a patient has a concomitant abdominal condition that must be treated surgically, surgery can be done to explore the area and drain the abscess [19]. 

Various variables that include a high daily drainage volume, a large abscess size, presence of gastrointestinal fistula, older age, and bacteriological factors can lead to failure of percutaneous drainage [20]. The presented patient did not have most of the listed variables but had a failed primary percutaneous abscess drainage that required surgical drainage and further percutaneous drainages. There could have been other factors not yet explored that need more research which may have contributed to the complication of the patient’s presentation. 

## Conclusions

The laparoscopic omental patch repair (Graham patch) is efficacious in treating perforations; however, this case study presented an uncommon occurrence of failed primary percutaneous abscess drainage, leading to subsequent surgical drainage. Following the clinical course of this patient, the initial diagnosis of intestinal perforation was done through CT, and an exploratory laparotomy was followed where a perforated gastric ulcer was found. The management of the perforation was through a Graham patch repair; and once leukocytosis was noticed to trend upward, an IV contrast study was done whereupon a formation of an abscess was confirmed. The case presentation emphasizes variables that can contribute to this complication, although the patient did not report the most common ones. When managing percutaneous abscess drainages, it is important to evaluate factors that both lead to the development of an efficient management plan and minimize abscess recurrence. In relation to this case, additional reported cases and prospective studies are needed to explore other factors not yet researched as well. 
